# Editorial: Systems immunology and computational omics for transformative medicine

**DOI:** 10.3389/fimmu.2025.1746667

**Published:** 2025-12-03

**Authors:** Aaron S. Meyer, Chuangqi Wang, Fan Zhang

**Affiliations:** 1 Department of Bioengineering, University of California Los Angeles, Los Angeles, CA, United States; 2 Department of Immunology and Microbiology, University of Colorado School of Medicine, Aurora, CO, United States; 3 Department of Biomedical Informatics, University of Colorado School of Medicine, Aurora, CO, United States; 4 Department of Medicine Rheumatology, University of Colorado School of Medicine, Aurora, CO, United States

**Keywords:** systems immunology, computational biology, translational medicine, omics, systems biology

Advances in single-cell, multi-omics, and spatial technologies now allow immunology to be studied at unprecedented resolution, but the complexity of these data demands dedicated computational approaches. Systems immunology addresses this need by integrating data-driven analysis with mechanistic insights to understand immune networks across scales. The papers of this Frontiers in Immunology Research Topic reflect two overarching themes within this evolving computational landscape.

The first theme reflects the innovative use of computational methods, multi-omics data integration, and systems-level thinking to unravel intricate biological mechanisms, particularly within immunology. The papers under this theme introduce novel algorithms, analytical pipelines, or theoretical frameworks to process large datasets and derive actionable insights from complex interactions at different biological scales.

Several papers exemplify this theme. Hanson et al. present a flexible systems analysis pipeline integrating cell-segmented imaging data with multivariate modeling to analyze spatial relationships within the tumor microenvironment and their relationship to tumor or patient features. Townsend et al. evaluate and benchmark computational methods for integrating scRNA-seq with GWAS to understand immune-mediated inflammatory diseases. Quiroz et al. develop a conceptual framework for viewing the immune system as a multiscale adaptive information processing network, using principles like self-organized criticality and antifragility to understand its operation.

The second theme reflects the expanded focus of computational and systems biology insights to real-world medical challenges, aiming to improve diagnostics, prognostics, and therapeutic strategies. The breadth of modern data collection and access necessitates computational techniques to identify biomarkers, predict patient outcomes, understand disease progression, and discover new therapeutic targets or interventions.

Focusing most closely on translational impact, Peng et al. introduce “Idbview,” a database and web platform centralizing clinical and omics data for respiratory diseases, aiming to facilitate biomarker identification and streamline research. Zhang et al. investigate the Systemic Immune-Inflammation Index as a biomarker for surgical invasiveness in robot-assisted pelvic fracture fixation, providing a clear example of informing clinical practice. Cui et al. utilize computational analysis of multi-omics and single-cell data to identify MHC-II-expressing Triple-Negative Breast Cancer (TNBC) cells and develop a prognostic gene signature with translational potential.

Systems approaches may also contribute to the interpretation and use of molecular characterization from the laboratory. Datta et al. developed a human *in vitro* model to study the tumor microenvironmental effects of Axl inhibition to inform clinical trial design. This intervention has complex and context-dependent effects that can only be understood through multivariate analysis. Liu et al. combine genetic analyses, multi-omics, and virtual screening to identify gut microbial metabolites and druggable targets, such as ILA binding to PFKFB2 for sepsis treatment, directly contributing to therapeutic development.

Collectively, the articles in this series highlight two complementary directions in systems immunology: the development of advanced computational frameworks to dissect immune complexity, and the translation of these insights into clinical contexts for biomarker discovery and therapeutic application. Continued progress will rely on close collaboration across computational, experimental, and clinical disciplines. Together, these studies demonstrate the growing impact of systems immunology in driving precision medicine.

